# *Acinetobacter* spp. are associated with a higher mortality in intensive care patients with bacteremia: a survival analysis

**DOI:** 10.1186/s12879-016-1695-8

**Published:** 2016-08-09

**Authors:** Aline C. Q. Leão, Paulo R. Menezes, Maura S. Oliveira, Anna S. Levin

**Affiliations:** 1 Department of Infectious Diseases and LIM 54, University of São Paulo, São Paulo, Brazil; 2 Department of Preventive Medicine, University of São Paulo, São Paulo, Brazil; 3 Infection Control Department, Hospital das Clínicas, University of São Paulo, São Paulo, Brazil; 4 Instituto de Medicina Tropical, University of São Paulo, São Paulo, Brazil

**Keywords:** *Acinetobacter*, Bacteremia, Intensive care units, Survival analysis, Prognosis

## Abstract

**Background:**

It has been challenging to determine the true clinical impact of *Acinetobacter* spp., due to the predilection of this pathogen to colonize and infect critically ill patients, who often have a poor prognosis. The aim of this study was to assess whether *Acinetobacter* spp. bacteremia is associated with lower survival compared with bacteremia caused by other pathogens in critically ill patients.

**Methods:**

This study was performed at Hospital das Clínicas, University of São Paulo, Brazil. There are 12 intensive care units (ICUs) in the hospital: five Internal Medicine ICUs (emergency, nephrology, infectious diseases and respiratory critical care), three surgical ICU (for general surgery and liver transplantion), an Emergency Department ICU for trauma patients, an ICU for burned patients, a neurosurgical ICU and a post-operative ICU. A retrospective review of medical records was conducted for all patients admitted to any of the ICUs, who developed bacteremia from January 2010 through December 2011. Patients with *Acinetobacter* spp. were compared with those with other pathogens (*Klebsiella pneumoniae*, *Staphylococcus aureus*, *Enterobacter* spp., *Enterococcus* spp., *Pseudomonas aeruginosa*). We did a 30-day survival analysis. The Kaplan-Meier method and log-rank test were used to determine the overall survival. Potential prognostic factors were identified by bivariate and multivariate Cox regression analysis.

**Results:**

One hundred forty-one patients were evaluated. No differences between patients with *Acinetobacter* spp. and other pathogens were observed with regard to age, sex, APACHE II score, Charlson Comorbidity Score and type of infection. Initial inappropriate antimicrobial treatment was more frequent in *Acinetobacter* bacteremia (88 % *vs* 51 %). Bivariate analysis showed that age > 60 years, diabetes mellitus, and *Acinetobacter* spp. infection were significantly associated with a poor prognosis. Multivariate model showed that *Acinetobacter* spp. infection (HR = 1.93, 95 % CI: 1.25–2.97) and age > 60 years were independent prognostic factors.

**Conclusion:**

*Acinetobacter* is associated with lower survival compared with other pathogens in critically ill patients with bacteremia, and is not merely a marker of disease severity.

## Background

It has been challenging to determine the true clinical impact of *Acinetobacter* spp., due to the predilection of this pathogen to colonize and infect critically ill patients, who often have a poor prognosis irrespective of secondary infective complications [1].

Some investigators found high mortality rates in intensive care unit (ICU) patients with *Acinetobacter* bacteremia: 61.6 % in Israel [2], 65.5 % in Brazil [3] and 43.4 % in the United States [4].

When outcomes from *Acinetobacter baumannii* were compared directly with those of patients who had bacteremia caused by other organisms, a significantly higher mortality was noted for *A. baumannii* [2, 5]. However, none of these studies used a formal, standardized method to adjust for severity of illness or comorbidities, such as APACHE or Charlson score. Another study involving trauma patients showed no difference in mortality comparing infections by *Acinetobacter* and by other pathogens [6]. Tonacio et al. [7] found 30 % of mortality in patients with *Acinetobacter* spp. infections and trauma was a marker of good prognosis in those patients.

Some studies observed growing resistance among other gram-negative and gram-positive pathogens that cause healthcare-associated infections. Rice [8] reported these as the “ESKAPE” pathogens: *Enterococcus faecium*, *Staphylococcus aureus*, *Klebsiella pneumoniae*, *Acinetobacter baumannii*, *Pseudomonas aeruginosa* and *Enterobacter* species [8, 9]. These pathogens cause an increasing number of healthcare-associated infections with significant morbidity and mortality, with are often associated with ICU admission [10].

The aim of this study was to evaluate whether bacteremia caused by *Acinetobacter* spp. was associated with lower survival compared with bacteremia caused by other prevalent pathogens in critically ill patients.

## Methods

This study was performed at Hospital das Clínicas, University of São Paulo, Brazil, a 2200-bed tertiary-care teaching hospital. There are 12 ICUs in the hospital; five Internal Medicine ICUs (emergency, nephrology, infectious diseases and respiratory critical care), three surgical ICU (for general surgery and liver transplantion), an Emergency Department ICU for trauma patients, an ICU for burned patients, a neurosurgical ICU and a post-operative ICU.

A retrospective review of medical records was conducted for all patients admitted to the ICUs who developed bacteremia from January 2010 through December 2011. The inclusion of the patients was based on notifications of nosocomial infections made by the Hospital Infection Control Team according to CDC/NHSN criteria [11]. All hospitalized patients with bacteremia by the selected pathogens were included in the study if the blood cultures were obtained > 48 h after admission to the ICU. In patients with recurrent bacteremia, only the first episode was included. Polymicrobial infections were excluded.

Patients with *Acinetobacter* spp. bacteremia were compared with patients with bacteremia caused by other pathogens (*Klebsiella pneumoniae*, *Staphylococcus aureus*, *Enterobacter* spp., *Enterococcus* spp., *Pseudomonas aeruginosa*). We selected these agents for comparison, as they were healthcare-associated pathogens of epidemiologic importance, had high antibiotic resistance rates, and were the predominant healthcare-associated pathogens in the hospital.

We evaluated prognostic factors associated with mortality. The following variables were assessed: sex; age; APACHE II score [12] on admission to ICU; use of invasive devices and antimicrobials after the diagnosis of bacteremia; initial site of infection and treatment; time elapsed from admission in the ICU to diagnosis of bacteremia; Pitt Bacteremia Score [12]; presence of septic shock; and number of organ failures. Acute organ failures (cardiovascular, respiratory, renal, hematologic or central nervous system) were defined using the definitions of Zimmerman et al. [13]. The patients’ underlying diseases analyzed were: diabetes mellitus, liver cirrhosis, cancer, transplant recipient, HIV infection, chronic renal disease, obstructive pulmonary disease, trauma, and systemic arterial hypertension. We also analyzed the Charlson Comorbidity Score [14].

Bacteremias were classified as primary and secondary bloodstream infections. Primary bloodstream infections were those associated with the use of a central line or those with an unknown or unclear initial site. Secondary bloodstream infections were regarded as those with a clear source of bacteremia other than a central line. Sources of secondary bacteremia were identified by cultures of samples (urine, tracheal secretions, intra-abdominal samples, etc.) obtained from distant sites that yielded the same pathogen with an identical resistance pattern. Distant sites were sites where an infection was diagnosed other than a central line (pneumonia, surgical site, urinary tract, skin and soft tissue, others).

Antibiotic treatment was deemed initial appropriate antibiotic treatment (IAAT) if the initially prescribed antibiotic regimen was active against the identified pathogen, based on in vitro susceptibility testing, and administered within two days following the blood culture collection. All other regimens were classified as initial inappropriate antibiotic treatment (IIAT).

### Microbiology

The clinical microbiology laboratory made the identification and antimicrobial susceptibility test of the selected pathogens using VITEK 2® (bioMerieux VITEK, Hazelwood, MO, USA). The breakpoints were those defined by the Clinical and Laboratory Standards Institute (CLSI) [15, 16].

The automatic identification method VITEK 2® (bioMerieux VITEK, Hazelwood, MO, USA) showed the results of *Acinetobacter* as *Acinetobacter baumannii-calcoaceticus* complex. This complex includes other pathogenic species besides *Acinetobacter baumannii, such* as *A. calcoaceticus, A. tjernbergiae (sp. 3), A. ursingii (sp.13).* As the isolates were not available for further identification, we chose to refer to the microorganism as *Acinetobacter* spp.

### Data analysis

We initially conducted a descriptive analysis comparing patients with *Acinetobacter* spp. bacteremia and patients with bacteremia caused by other pathogens. Baseline characteristics and outcomes were described using summary (mean, standard deviation, median, minimum and maximum) for quantitative variables and absolute and relative frequencies for qualitative variables.

We did a 30-day survival analysis. For overall survival time, we estimated median survival time according to the characteristics of interest using the Kaplan-Meier function and compared survival rates among the categories using the log-rank test. The bivariate Cox regression was chosen to calculate the hazard ratio (HR) in survival analysis, with a 95 % confidence interval.

It was estimated the multiple Cox regression model with the variables with descriptive level in bivariate tests less than 0.10 (*p* <0.10) and considered with biological plausibility. The tests were done at 5 % significance level. In the case of variables that we considered measured similar characteristics, only one variable was included in the model. Statistical analyses were performed using SPSS (Version 19.0).

## Results

Three hundred forty-nine patients presented with the selected pathogens bacteremia during the 2-year study period (128 *Acinetobacter* spp., 55 *Klebsiella pneumoniae*, 40 *Pseudomonas aeruginosa*, 33 *Enterobacter* spp., 49 *Staphylococcus aureus* and 44 *Enterococcus* spp.). 208 were excluded (99 had previous positive blood cultures, 68 had polymicrobial bacteremia, 27 had blood cultures obtained ≤ 48 h after admission in the ICU, nine had incomplete records and five had unavailable records). Thus 141 patients were evaluated (59 with *Acinetobacter* spp. bacteremia and 82 with bacteremia caused by other pathogens). The other pathogens were *K. pneumoniae* (n: 24), *S. aureus* (n: 21), *Enterobacter* spp. (n: 15), *Enterococcus* spp. (n: 12) and *P. aeruginosa* (n: 10).

Patient characteristics by pathogen are detailed in Table 1. No differences between *Acinetobacter* spp. and other pathogens were observed with regard to age, sex, APACHE II score, Charlson Comorbidity Score, duration of hospitalization in the ICU prior to bacteremia and initial site of infection. A detailed analysis of background disease demonstrated no difference between the two groups of patients. Chronic diseases were frequent, including systemic arterial hypertension, cancer, chronic renal disease, diabetes mellitus, solid organ transplants, liver cirrhosis, trauma, obstructive pulmonary disease, HIV infection and hematopoietic stem cell transplantation.

**Table 1:** General characteristics of the entire cohort of patients with bacteremia acquired in intensive care units

	*Acinetobacter* spp.	Other pathogens^a^	Total
Number of patients (%)	59 (42)	82 (58)	141 (100)
Age			
Mean (SD)	52 (18)	56 (16)	54 (17)
Median (overall range)	51 (17–92)	57 (18–85)	56 (17–92)
Male sex (%)	42 (71)	45 (55)	87 (62)
APACHE II score			
Mean (SD)	20 (7)	20 (9)	20 (8)
Median (overall range)	20 (7–40)	19 (0–41)	19 (0–41)
CHARLSON score			
Mean (SD)	3 (3)	3 (3)	3 (3)
Median (overall range)	2 (0–10)	3 (0–11)	3 (0–11)
Co-morbid condition (%)			
Diabetes mellitus	14 (24)	17 (21)	31 (22)
Liver cirrhosis	18 (31)	10 (12)	28 (20)
Cancer	12 (20)	21 (26)	33 (23)
Solid organ transplant	17 (29)	13 (16)	30 (21)
Liver transplant	14 (24)	10 (12)	24 (17)
Kidney transplant	3 (5)	3 (4)	6 (4)
Hematopoietic cell transplant	0 (0)	2 (2)	2 (1)
HIV infection	3 (5)	6 (7)	9 (6)
Chronic renal disease	15 (25)	16 (20)	31 (22)
Obstructive pulmonar disease	4 (7)	8 (10)	12 (9)
Trauma	8 (14)	7 (9)	15 (11)
Systemic arterial hypertension	19 (32)	37 (45)	56 (40)
ICU length of stay previous to bacteremia (in days)			
Mean (SD)	11 (14)	17 (36)	15 (29)
Median (overall range)	7 (2–82)	9 (2–314)	8 (2–314)
Initial site of infection			
Bloodstream	43 (73)	58 (71)	101 (72)
Pneumonia	3 (5)	11 (13)	14 (10)
Surgical site	5 (8)	6 (7)	11 (8)
Urinary tract	1 (2)	2 (2)	3 (2)
Skin and soft tissue	3 (5)	0 (0)	3 (2)
Other	4 (7)	5 (7)	9 (6)

*SD* standard deviation, *ICU* intensive care unit

^a^Includes *Klebsiella pneumoniae* (n: 24), *Staphylococcus aureus* (n: 21), *Enterobacter* spp. (n: 15), *Enterococcus* spp. (n: 12), *Pseudomonas aeruginosa* (n: 10)

Both groups of pathogens presented high rates of resistance to antibiotics. Most *Acinetobacter* spp. were resistant to carbapenems (92 %) and susceptible to colistin (95 %). Among the other pathogens, resistance to methicillin was 71 % among *Staphylococcus aureus*; among *Enterococcus* spp. 83 % were vancomycin-resistant (VRE); and carbapenem resistance in *Pseudomonas aeruginosa* was 30 %; 27 % in *Klebsiella pneumoniae* and 7 % in *Enterobacter* spp. isolates.

**Fig. 1:**
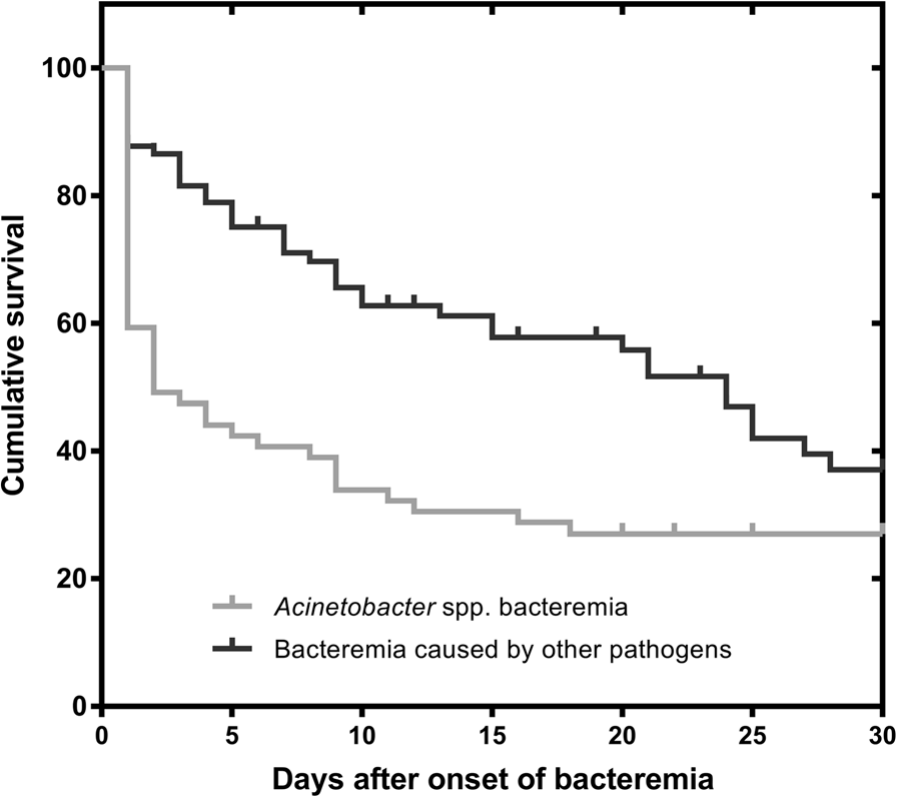
Cumulative survival after episodes of *Acinetobacter* spp. bacteremia and bacteremia caused by other pathogens. The curve was illustrated with the Kaplan-Meier method (log-rank test, *p* = 0.005)

**Fig. 2:**
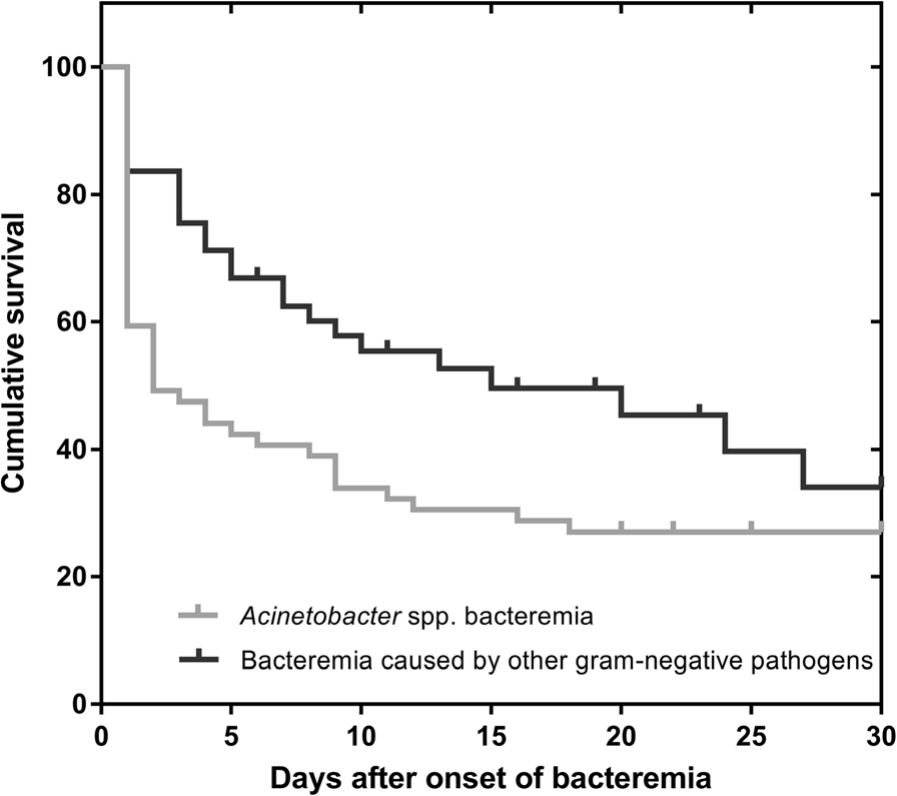
Cumulative survival after episodes of *Acinetobacter* spp. bacteremia and bacteremia caused by other gram-negative pathogens. The curve was illustrated with the Kaplan-Meier method (log-rank test, *p* = 0.033)

Initial inappropriate antibiotic treatment was administered to 88 % of patients with *Acinetobacter* spp. and 51 % of patients with other pathogens. More patients with *Acinetobacter* spp. developed septic shock (81 % *vs* 52 %); needed mechanical ventilation within 24 h of the diagnosis of bacteremia (88 % *vs* 66 %); and required a central venous line (97 % *vs* 85 %). Patients with *Acinetobacter* spp. bacteremia had a higher mortality when compared with bacteremia by the other pathogens (73 % *vs* 50 %). The mean Pitt Bacteremia Score for *Acinetobacter* spp. was 7 (SD: 4) and for other pathogens was 4 (SD: 3). The mean number of organ failures for *Acinetobacter* spp. was 2.1 (SD: 1.2) and for other pathogens was 1.67 (SD: 1.3). The cumulative survival curves of the patients according to pathogen are shown in Figs. 1 and 2.

The bivariate analysis (Table 2) showed that age >60 years, *Acinetobacter* spp. infection, and diabetes mellitus were significantly associated with a poor prognosis. The following variables also presented *p* < 0.10 in the bivariate analysis: sex; liver cirrhosis; obstructive pulmonary disease, and IIAT. The variables: number of organ failures; septic shock; Pitt Bacteremia Score (which evaluates the severity of the bacteremia), mechanical ventilation and use of central venous line were excluded from the bivariate and multivariate analyses because they were considered intrinsically correlated with the event death and not proper prognostic factors. We verified that these factors, excluded from the multivariate analysis, were statistically associated with the outcome, except for use of central venous line (data not shown). Most patients with diabetes mellitus were older than 60 years, thus the variable diabetes mellitus was also not included in the multivariate analysis. Among the cases of bacteremia by *Acinetobacter* spp. most received IIAT (88 %) thus we did not enter this variable into the model. Thus, in the model of Cox regression analysis we evaluated the following variables: age divided into the following strata: ≤ 60 years or > 60 years; sex; liver cirrhosis; obstructive pulmonary disease; and *Acinetobacter* spp. bacteremia. The multivariate model showed that *Acinetobacter* spp. infection (HR: 1.93, 95 % CI 1.25–2.97) and age > 60 years were statistically associated with mortality (Table 3).

**Table 2:** Bivariate analysis of prognostic factors of patients with bacteremia acquired in intensive care units

Variables	Median survival time (days)	95 % CI	HR	95 % CI	Death/total (%)	*P*
Age > 60 years						
Yes	5	0.00–10.82	1.67	1.08–2.58	37/53 (70)	0.02
No	20	11.07–28.93	1		47/88 (53)	
Sex						
Male	9	4.32–13.69	1.49	0.95–2.35	56/87 (64)	0.08
Female	25	9.67–40.34	1		28/54 (52)	
APACHE II Score > 20						
Yes	10	3.48–16.52	1.11	0.72–1.70	39/60 (65)	0.64
No	15	5.10–24.9	1		45/81 (56)	
CHARLSON Score > 3						
Yes	15	1.78–28.22	1.18	0.76–1.84	31/48 (65)	0.46
No	11	5.32–16.68	1		53/93 (57)	
Co-morbid condition						
Diabetes mellitus						
Yes	7	^a^	1.67	1.03–2.70	23/31 (74)	0.03
No	16	6.86–25.14	1		61/110 (55)	
Liver cirrhosis						
Yes	5	0.01–9.99	1.58	0.96–2.59	21/28 (75)	0.07
No	16	4.30–27.70	1		63/113 (56)	
Cancer						
Yes	18	0.00–39.72	0.87	0.52–1.46	18/33 (55)	0.59
No	10	5.01–14.99	1		66/108 (61)	
Solid organ transplant						
Yes	5	0.98–9.02	1.31	0.80–2.15	21/30 (70)	0.28
No	16	7.22–24.78	1		63/111 (57)	
Hematopoietic stem cell transplant						
Yes	7	^a^	0.87	0.12–6.26	1/2 (50)	0.89
No	11	4.21–17.79	1		83/139 (60)	
HIV infection						
Yes	^a^	^a^	0.61	0.22–1.66	4/9 (44)	0.33
No	10	4.12–15.88	1		80/132 (61)	
Chronic renal disease						
Yes	10	0.56–19.45	1.30	0.79–2.14	21/31 (68)	0.29
No	13	3.92–22.08	1		63/110 (57)	
Obstructive pulmonary disease						
Yes	8	0.00–21.58	1.73	0.92–3.26	11/12 (92)	0.09
No	12	3.85–20.15	1		73/129 (57)	
Trauma						
Yes	^a^	^a^	0.57	0.25–1.30	6/15 (40)	0.18
No	10	3.27–16.73	1		78/126 (62)	
Systemic arterial hypertension						
Yes	8	2.39–13.61	1.16	0.75–1.80	33/56 (59)	0.51
No	15	6.39–23.61	1		51/85 (60)	
ICU length of stay previous to bacteremia > 8 days						
Yes	20	8.90–31.10	0.78	0.51–1.21	37/65 (57)	0.27
No	9	5.20–12.80	1		47/76 (62)	
Initial site of infection						
Primary bloodstream						
Yes	13	4.71–21.29	1.01	0.63–1.62	60/101 (59)	0.97
No	9	2.98–15.02	1		24/40 (60)	
Pneumonia						
Yes	8	0.67–15.33	1.32	0.68–2.56	10/14 (71)	0.41
No	13	4.88–21.13	1		74/127 (58)	
Surgical site						
Yes	13	4.22–21.78	1.22	0.58–2.52	76/130 (58)	0.60
No	9	3.53–14.47	1		8/11 (73)	
Urinary tract						
Yes	^a^	^a^	0.05	0.00–10.63	0/3 (0)	0.27
No	^a^	^a^	1		84/138 (61)	
Skin and soft tissue						
Yes	1	^a^	1.40	0.34–5.71	2/3 (67)	0.64
No	12	5.27–18.73	1		82/138 (59)	
*Acinetobacter* spp.						
Yes	2	0.00–4,51	1.85	1.21–2.85	43/59 (73)	0.005
No	24	19.42–28,58	1		41/82 (50)	
IIAT						
Yes	9	4.46–13.54	1.53	0.98–2.36	50/73 (68)	0.057
No	24	7.06–40.95	1		34/68 (50)	

*CI* Confidence interval, *HR* Hazard Ratio, *ICU* intensive care unit, *IIAT* initial inappropriate antimicrobial treatment

^a^Not possible to calculate median time and confidence interval

**Table 3:** Multivariate model of prognostic factors of patients with bacteremia acquired in intensive care units

	Crude HR	95 % CI	Adjusted HR	95 % CI	*P*
*Acinetobacter* spp.					0.003
No	1		1		
Yes	1.85	1.21–2.85	1.93	1.25–2.97	
Age					0.012
≤ 60 years	1		1		
> 60 years	1.67	1.08–2.58	1.75	1.13–2.70	

*CI* Confidence interval, *HR* Hazard Ratio

## Discussion

Our study was conducted to evaluate prognostic factors, especially *Acinetobacter* spp. infection, in patients with bacteremia acquired in ICU. We concluded that patients who had *Acinetobacter* spp. bacteremia presented a significantly worse prognosis, independently of severity of the clinical condition and other potential confounders. Another important aspect was the short period of time between *Acinetobacter* bacteremia and death.

The increase in the number of infections caused by multidrug-resistant bacteria, especially gram-negative bacilli, is one of the most important issues in modern healthcare [17]. Among several gram-negative bacilli, non-fermentative organisms such as *Pseudomonas aeruginosa* and *Acinetobacter baumannii* are the most problematic because of their high frequency and wide spectrum of antimicrobial resistance. This leads to a limited therapeutic armamentarium against them [18, 19]. At our hospital, from January, 2010 through December 2011, 14 % of all episodes of bacteremia were polymicrobial. Of all monomicrobial episodes, most were caused by gram-negative organisms. The rank order of the major pathogens shows that *Acinetobacter* spp. were the principal organisms responsible for bacteremias (22 %), and most of *Acinetobacter* spp. were resistant to carbapenems.

Administering appropriate initial antibiotic therapy is essential in the treatment of septic patients [20] and is associated with lower mortality rate in patients with *Acinetobacter* spp. bacteremia [21, 22]. Our study found that 92 % of the *Acinetobacter* spp. isolates were carbapenem-resistant and, in most cases, colistin was the only available antimicrobial agent to treat these serious infections. The time required for identification of *Acinetobacter* spp. by culture and for identifying carbapenem resistance was greater than the maximum time (48 h) defined in the present study for beginning the appropriate therapy.

Without microbiological information as a guide, only 12 % of patients with *Acinetobacter* spp. bacteremia received effective drugs within 48 h, possibly contributing to the high mortality rate in these patients. In the bivariate analysis of prognostic factors, IIAT appears to be associated with mortality, with a borderline significance (*p* = 0.057). In the multivariate model, *Acinetobacter* was associated with poor prognosis, but IIAT may have a part in explaining why *Acinetobacter* cases had a worse prognosis.

Our data show the high mortality of infections caused by carbapenem-resistant *Acinetobacter* spp. Based on our findings, we suggest that early initiation of treatment including colistin is important to improve survival in ICUs where infections by these isolates are frequent. Our results also suggest the need for more effective antibiotic stewardship programs to avoid unnecessary treatment with broadly active antibacterial therapy that selects for carbapenem resistance. New infection prevention strategies and technologies are needed against these infections.

Some studies suggest that *Acinetobacter* spp. are opportunistic pathogens that affect patients who are more likely to die because of the severity of their prior disease [23–25]. Blot et al. [26] compared *Acinetobacter baumannii* bacteremia with matched controls and found that *Acinetobacter baumannii* was not an independent predictor for mortality. In another single-center experience [6], *Acinetobacter baumannii* infection, including multidrug-resistant isolates, the impact on mortality in a cohort of trauma patients was not conclusive. However, *Acinetobacter baumannii* infection was associated with a longer intensive care unit stay and a higher rate of organ failures.

In a review article, Peleg et al. [1] showed that the studies on prognosis of *Acinetobacter* infections lacked an adequate evaluation of the patients’ severity of underlying condition. Thus, in our study, we used formal and standardized methods to adjust for severity of illness and comorbidities (APACHE II and Charlson Score). Surprisingly, these variables and the underlying diseases were not significant prognostic factors. These findings support that the high mortality caused by this serious healthcare-associated pathogen cannot be attributed only to underlying conditions and that *Acinetobacter* infections are not merely markers of the severity of the patients’ clinical condition.

The median survival of the *Acinetobacter* group was only two days, thus suggesting the severity of the infection. In our study, the median of Pitt Bacteremia Score was higher in the *Acinetobacter* spp. group. Rhee et al. suggested that the Pitt bacteremia score is an excellent tool for assessing not only crude mortality, but also mortality that is attributed to sepsis in ICU-admitted patients [12].

Some investigators found high mortality rates in ICU patients with *Acinetobacter* bacteremia (43.4 to 61.6 %) [2–4]. Virulence factors and genotypes of *Acinetobacter* may have an important role in differences in mortality. Few clinical data are available on the relationship between genospecies and outcome of *Acinetobacter* bacteremia. Park et al. [27] compared the clinical features, antimicrobial resistance, and outcome of bacteremia caused by *Acinetobacter baumannii* versus non-*baumannii* of the *Acinetobacter calcoaceticus–baumannii* (ACB) complex. The study found that the species, rather than the antibiotic resistance, affected mortality, in accordance with other studies [28, 29]. Peleg et al. suggested that in vitro and in vivo virulence characteristics differed among individual strains of the ACB complex [30], which provides further evidence of the impact of genospecies on the outcome of *Acinetobacter* bacteremia. In our retrospective study, we could not identify these factors, but the evaluation of species and virulence factors in future epidemiological and clinical studies of *Acinetobacter* infections may be important.

Several limitations of this study are noteworthy. Because it is a single-center study, our findings may be attributable to institution-specific variables and may not reflect the epidemiology of different centers or geographical areas. The study was retrospective and some patients were excluded because of incomplete data. Molecular identification of the isolates was not performed to identify the genomic species of *Acinetobacter*.

## Conclusions

Our study adds to the existing evidence and the results support that *Acinetobacter* is associated with lower survival compared with other pathogens in critically ill patients with bacteremia, and is not merely a marker of disease severity.

## Abbreviations

ACB, *Acinetobacter calcoaceticus–baumannii*; CLSI, Clinical and Laboratory Standards Institute; IAAT, initial appropriate antibiotic treatment; ICU, intensive care unit; HR, hazard ratio; SD, standard deviation; VRE, vancomycin-resistant *Enterococcus*
